# COVID-19 and chronic fatigue syndrome: An endocrine perspective

**DOI:** 10.1016/j.jcte.2021.100284

**Published:** 2021-12-03

**Authors:** Rashika Bansal, Sriram Gubbi, Christian A. Koch

**Affiliations:** a National Institute of Diabetes and Digestive and Kidney Diseases, National Institutes of Health, Bethesda, MD, United States; b Department of Medicine, Fox Chase Cancer Center, Philadelphia, PA, United States; c Dept of Medicine/Endocrinology, The University of Tennessee Health Science Center, Memphis, TN, United States

**Keywords:** Long Covid, COVID-19, Chronic Fatigue Syndrome, Endocrinopathy, Adrenal function, Thyroid, Pituitary, Pain

## Abstract

Patients recovering from COVID-19 may have persistent debilitating symptoms requiring long term support through individually tailored cardiopulmonary and psychological rehabilitation programs. Clinicians need to be aware about the likely long-term complications and their diagnostic assessments to help identify any occult problems requiring additional help. Endocrinological evaluations should be considered as part of the armamentarium in the management of such individuals with diligent cognizance about the involvement of the hypothalamo-pituitary-adrenal (HPA) axis, adrenal and thyroid function. We here review the literature and potential pathophysiological mechanisms involved in and related to post COVID-19 symptoms with an emphasis on endocrine function.

## Introduction

At the end of 2019, a novel coronavirus termed severe acute respiratory syndrome coronavirus type 2 (SARS-CoV-2) was identified as the cause of a cluster of pneumonia cases in Wuhan, China. Since then, this virus has spread rapidly and caused a pandemic that wreaked havoc at a global level. In 2015, Menachery and colleagues already (pre-SARS-CoV-2) presented evidence for a potential risk of SARS-CoV re-emergence from viruses circulating in bat populations [1].

COVID-19 is considered a multiorgan disease and as the complications caused by COVID-19 continue to unfold, it is becoming evident that there is a section of people who despite having recovered from acute effects of the COVID-19 illness, continue to suffer from persisting and cyclical symptoms. Based on the COVID-19 Symptom Study, a study carried-out on more than 4 million people in the United States, United Kingdom, and Sweden wherein people entered their ongoing symptoms on a smartphone app, around 10% of patients who have tested positive for SARS-CoV-2 virus remain symptomatic beyond 3 weeks, and a smaller fraction for months [2].

This spectrum of persisting symptoms, ranging from mild to debilitating, is being termed as Post-COVID syndrome or Long COVID and can have variable presentation in people irrespective of the severity of their initial disease. Davis et al. analyzed 3762 participants, from 56 countries, with confirmed (N = 1020) or suspected (N = 2742) COVID-19 and found that 91% of respondents took more than 35 weeks to recover [3]. Forty-five percent of the patients required a reduced work schedule compared to pre-illness, and 22.3% were not working at 7 months follow up due to illness [3]. Most common symptoms include extreme tiredness, shortness of breath, brain fog, changes to taste and smell, joint pains etc. Surveys have identified hundreds of complaints. Chronic fatigue syndrome (CFS) remains central and the most common complaint in patients, restricting their daily activities like showering, grocery shopping, or walking.

**Fig. 1:**
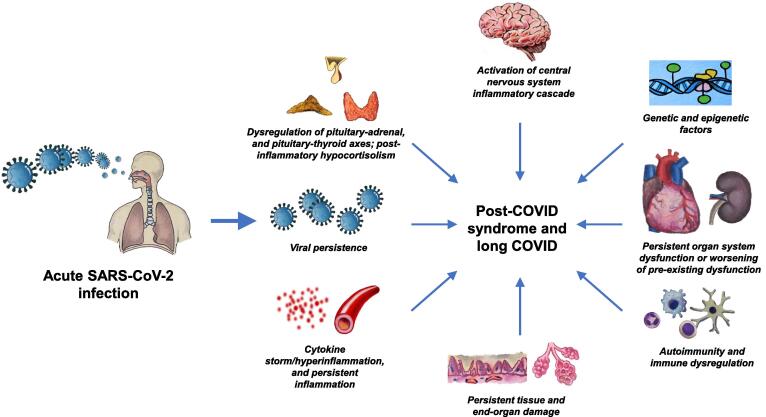
Potential pathophysiological mechanisms involved in the development of post-COVID syndrome and long COVID (Image courtesy: Sriram Gubbi, NIDDK, NIH).

It is unknown what causes long COVID and CFS. Whether it is continued inflammatory or autoimmune responses, or continued damage by the reactivated virus residues, yet needs to be elucidated (Fig. 1).

The role of endocrine disorders in the causation of long-term symptoms post-COVID especially CFS is not fully understood. Since endocrinopathies are treatable it is essential to identify any contribution they may have to the persistent symptoms experienced by patients after COVID-19 infection.

## Clinical features of COVID-19

The spectrum of clinical presentation of COVID-19 varies from asymptomatic to severe illness. Asymptomatic cases of COVID-19 have been well documented [4], [5], [6]. A large *meta*-analysis reported one in six cases of COVID-19 as asymptomatic with lower risk of transmission [7]. Another review based on studies in England (N = 365 104) and Spain (n = 61075) suggested that at least one third of SARS-CoV-2 infections were asymptomatic [8]. The symptomatic cases also have a wide range of presentation usually determined by risk factors such as age, sex, existence of comorbidities, socioeconomic background, genetic factors, the viral variants or laboratory abnormalities. The symptoms in most cases present within 4–5 days after exposure with incubation periods extending up to 14 days. In a pooled analysis, the median incubation period was estimated to be 5.1 days (95% CI, 4.5 to 5.8 days), and 97.5% developed symptoms within 11.5 days (95% CI, 8.2 to 15.6 days) of infection [9]. The initial manifestations of COVID-19 can include either one or two prominent symptoms or entire gamut of flu-like features such as cough, headaches, myalgias, sore throat, nausea/vomiting, diarrhea, fever, confusion, fatigue etc. Other symptoms such as loss of smell or taste are also well described [10], [11]. In a report describing outcomes among 1,320,488 laboratory-confirmed COVID-19 cases reported to the CDC during January 22 and May 30, 2020, the most common symptoms were cough (50%), fever (43%), myalgia (36%), headache (34%) among others with loss of smell or taste in fewer than 10% of subjects [12]. Development of pneumonia marks a step up in severity of illness of COVID-19 and presents as cough, fever, dyspnea and appearance of lung infiltrates. Some patients with initial non-severe presentation may worsen to develop critical illness with complications such as respiratory failure, thromboembolic and cardiovascular complications, neurological complications, secondary infections and other inflammatory sequelae. The recovery time from COVID-19 is also highly variable and depends on the severity of disease, age and preexistence of comorbidities. Individuals with mild infection can have a recovery time as quick as a few days to within two weeks, whereas individuals with severe disease can have a longer recovery time of 2 to 3 months. If symptoms continue beyond 4 weeks since onset of infection, the term long COVID has been used, whereas persistence of symptoms for more than 12 weeks has been named “Post-COVID syndrome” [13].

### Long COVID

As witnessed during previous global epidemics of severe acute respiratory syndrome coronavirus (SARS-CoV) and Middle East respiratory syndrome coronavirus (MERS-CoV), existence of long-term complications and persisting debilitating symptoms with current COVID-19 is not a novel occurrence. Tansley et al. reported 18% of individuals (n = 117) with SARS-CoV infection had a reduced 6-minute walk test due to shortness of breath and fatigue after 1 year as well as reduced quality of life at 3 months, with improvement but not normalization after one year [14]. Batawi et al. similarly followed 78 MERS-CoV survivors and found that their quality of life was reduced, with significantly lower quality in those who had had critical care admissions [15].

### Definition

Post-COVID syndrome and long COVID are evolving secondary syndromes in which patients recovering from SARS-CoV-2 suffer from persistent symptoms that extend several months after their initial diagnosis. The National Institute of Clinical Excellence (NICE) differentiates the two terms: post-COVID syndrome and long COVID. It defines post-COVID syndrome as signs and symptoms that develop during or after an infection consistent with COVID–19 and continue for more than 12 weeks, that are not explained by an alternative diagnosis [16]. The other term long COVID is commonly used to characterize both ongoing symptomatic COVID–19 (from 4 to 12 weeks) and post–COVID–19 syndrome (12 weeks or more) [16].

### Prevalence, risk factors and pathophysiology

Knowledge about the prevalence, pathology, predictors or risk factors of developing long COVID-19 remains sparse. Based on data of the COVID-19 symptom study, the chances of developing long COVID are affected by age with rates being 1 to 2 % in patients in their twenties to about 5% in people in their sixties [2]. In a prospective cohort study in Bangladesh, among 46% patients (n = 400) who developed post-COVID-19 symptoms, post viral fatigue was the most common symptom (70% cases) and patients with female gender, respiratory distress, longer duration of disease and lethargy were more susceptible to developing post-COVID syndrome [17]. The heterogeneity of long COVID with respect to symptom duration, frequency, initial disease severity and patient characteristics makes it highly unpredictable. Shah et al. identified fatigue and persisting shortness of breath as the most frequent symptoms of long COVID [18]. The spectrum of symptomatology also includes cough, headache, myalgia, cognitive and mental disorders, chest and joint pains, smell and taste dysfunctions, insomnia, wheezing, rhinorrhea, sputum, and cardiac and gastrointestinal issues that may persist for six months after their onset. The frequency of prevalence of various symptoms in long COVID has been summarized in Table 1 [19], [20], [21], [22], [23], [24], [25].

**Table 1:** **Prevalence of Long-COVID Symptoms**[19], [20], [21], [22], [23], [24], [25].

**Very often**	**Often**	**Rare**
Fatigue	Sleep disturbance	Palpitations
Reduced energy level	Anxiety and depressive mood	Nausea
Dyspnea	Cognitive Problems	Dizziness
Headache	Generalized pain	Diarrhea
Dysfunctional sense of smell and taste	Hair loss	Dys-and paresthesia
	Cough	

Several hypothesis have been suggested to understand the pathophysiology of post-COVID syndrome relating to hyperinflammatory states, oxidative stress, cytokine storm and DNA damage [26]. A 3-month follow-up study showed pulmonary radiological abnormalities persisted in 70% of the subjects [27]. Similarly, abnormal lung functions as well as structural changes were reported to in mild-to- critical COVID-19 patients for up to 6 months [28], [29], [30]. Myocardial inflammation and cardiac abnormalities were found respectively in 60% and 78% subjects, independent of their pre-existing disease severity, in a German follow up study of 100 COVID-19 patients [31]. Yong S. reviewed literature on long COVID and suggested that the potential pathology for long term COVID to be / lie in persistent pulmonary, neurological or cardiac tissue damage as well as viral load mediated inflammation and immune dysregulation [32].

### Long-COVID and chronic fatigue syndrome

Myalgic encephalomyelitis/ Chronic Fatigue Syndrome (ME/CFS) is a term given to a gamut of symptoms such as fatigue, post exertional malaise, sleep disturbances, cognitive impairment, and non-provoked pain that persist for more than 6 months with substantial intensity and not completely explained by any medical condition. It is a heterogeneous condition with a multifactorial etiology involving immune, virologic, psychological, endocrine and other factors. Fatigue is known to occur after many virus infections or other infectious agents, the most prominent example being the Epstein-Barr virus [33].

An uncanny resemblance has been observed between the long-COVID syndrome and clinical features of CFS, though this is not an unfamiliar concept. Similar to ME/CFS, fatigue, myalgia, depression and poor sleep were seen in a cohort of 22 patients and a post-SARS-CoV syndrome [34]. Likewise, CFS like symptoms were described in 48% of survivors of MERS-CoV at 1 year [35]. A systematic review of published manuscripts on Long-COVID found that 55.17% of reports were mainly based on chronic fatigue and pain as main symptoms [36]. Multiple other studies done across the world showed chronic fatigue as the most frequent persisting symptom of long-COVID, irrespective of initial COVID-19 presentation severity or presence of respiratory distress [17], [21], [37], [38], [39]

### Factors contributing to fatigue after COVID-19

An important feature of viruses linked to ME/CFS is the ability to establish persistent and chronic infections. The symptom of chronic fatigue could be caused by damage to multiple organ systems during the COVID-19 disease leading to impairment of heart, lung, or kidney function. Of note, patients with severe COVID-19 including severe lung involvement can completely recover. An overall state of inflammation along with increase in inflammatory mediators as well as activation of cell-mediated immunity could possibly be contributing to the CFS-like state. Patients with long/post-COVID syndrome are predominantly female analogous to other autoimmune disorders and experts postulate a T- and B-cell dysregulation, possibly changes in the microbiota of the gastrointestinal tract [40], [41]. Lymphopenia is a typical feature of severe COVID-19 and T- and/or B-cell deficiency correlates with persistent/ongoing virus shedding [42], [43]. How much molecular mimicry plays a role in the pathogenesis of autoimmune phenomena related to COVID-19 is unknown. In people without chronic cardiac, pulmonary or renal dysfunction, a state of chronic low-grade neuroinflammation created by the SARS-CoV-2 virus can be one potential explanation for the chronic fatigue [44]. Disruption of routine life due to continuation of debilitating symptoms after COVID-19, social isolation as well as post traumatic syndrome caused by severe illness requiring mechanical ventilation can lead to depression which in turn can trigger CFS. Furthermore, endocrine dysfunctions leading to hypocortisolism, hypothyroidism or hypothalamo-pituitary-adrenal (HPA) axis disruption can be other potential explanations for CFS, though long term studies are warranted to further elucidate their role.

### The endocrine connection to Long-COVID syndrome

**Fig. 2:**
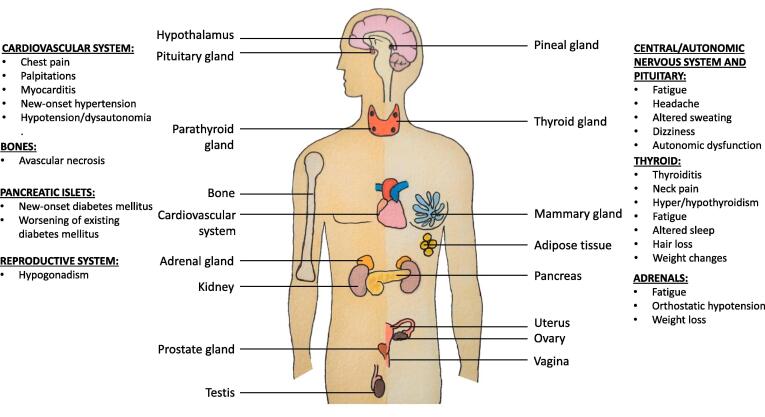
Manifestations of long-COVID on the endocrine system (Image courtesy: Sriram Gubbi, NIDDK, NIH).

ACE2 receptors, the route of entry of the SARS-CoV-2 virus into the human body, are expressed (https://www.proteinatlas.org/ENSG00000130234-ACE2/tissue) in the hypothalamus, pituitary, adrenal gland, thyroid, testes, and pancreatic islets leading to the involvement of the endocrine system during and after the recovery of the disease. Longitudinal and postmortem studies conducted on SARS-CoV patients provide some guidance on the extent of endocrine gland involvement. On postmortem examination, SARS-CoV RNA was found in the pituitary gland, parathyroid, pancreas and adrenal gland [45]. In another study, both parafollicular and follicular cells were found to be apoptotic explaining the low serum triiodothyronine and thyroxine levels and the osteonecrosis of the femoral head associated with patients of SARS-CoV [46]. Evidence of hypocortisolism was found in 39% of sixty-one survivors of SARS prospectively recruited for hormonal derangements 3 months after recovery [47]. Nonetheless, information about the adverse effects on endocrine function by the SARS-CoV-2 virus remains limited [48], [49]. The manifestations of long-COVID syndrome due to endocrine gland involvement have been depicted in Fig. 2.

### HPA axis

In autopsies of patients who died of SARS infection, evidence of viral genome, edema, and neuronal degeneration has been found in the hypothalamus [49]. In another study on recovered patients from previous SARS infection, hypocortisolism persisted for up to 1 year in the majority of patients along with central hypothyroidism and low dehydroepiandrosterone sulfate/DHEAS in some patients, supporting chronic corticotropin (ACTH) deficiency [47]. The authors proposed hypothalamo-pituitary dysfunction could be either from reversible hypophysitis or a direct hypothalamic damage [47]. Further, Wheatland et al. showed that the SARS virus expresses amino acids that mimic ACTH residues [49], [50]. The antibodies produced by the host as a response to the virus may cross-react with ACTH, unknowingly destroying it and lead to relative ACTH deficiency. Tertiary adrenal insufficiency due to abrupt cessation of high dose glucocorticoid treatment, leading to reduced corticotropin-release hormone and in turn decreased ACTH is another probable mechanism of HPA axis disruption.

However, studies on HPA axis involvement in post COVID-19 remain limited. A study evaluating the adrenocortical response in acute COVID-19 in 28 hospitalized patients found 32% patients had subnormal cortisol levels and more severe disease patients had both subnormal cortisol and ACTH levels, suggesting a direct association between the degree of COVID-19 infection and impaired glucocorticoid response [51]. Theoretically, hypothalamic and pituitary tissues do express ACE2 and can be viral targets for long term damage [52], however, prospective studies on HPA axis involvement for prolonged duration are needed. It is noteworthy that in CFS patient groups, blunting of the HPA axis, with reduced 24-hour free cortisol excretion, increased sensitivity to ACTH and attenuated response to CRH has been found [53]. Patients with long-COVID complaining of unexplained fatigue, lassitude, malaise, orthostatic dizziness, anorexia and apathy especially along with features of hypothyroidism, not resolving with hydration and traditional treatments, should be suspected of having a dysfunctional HPA axis and clinicians should have a low threshold to test HPA functionality.

### Adrenal gland

Due to non-specific clinical symptoms such as abdominal pain, vomiting, fever, fatigue, hypotension, and confusion, diagnosis of adrenal insufficiency as a cause of ongoing disease presentation of long-COVID is rarely suspected or tested. Autopsy studies in the previous outbreak of SARS showed that the cells undergo necrosis and identified the virus in the adrenal cortical cells suggesting a direct deleterious effect of the virus [54], [55]. Similarly, postmortem studies and several case reports on COVID-19 patients have reported microscopic adrenal lesions [56], [57], adrenal hemorrhage [58], [59], as well as adrenal infarction [60] with subsequent primary adrenal insufficiency. Of note, in the postmortem study by Santana et al., microscopic adrenal lesions were identified in 46% (N = 28) of patients; however, on cortisol measurement of stored samples of plasma collected 1 or 2 days before death, no adrenal insufficiency was found. Further, it has been established through the RECOVERY trial that in patients hospitalized with COVID-19, the use of dexamethasone resulted in lower 28-day mortality among those who were receiving either invasive mechanical ventilation or oxygen alone at randomization [61]. Due to rampant use of glucocorticoids in the treatment of COVID-19, apart from cytopathological damage, occurrence of iatrogenic adrenal insufficiency ought to be considered a possibility also.

On the contrary, Clarke et al. conducted a prospective study in 70 COVID-19 patients and found that adrenal and thyroid function ≥ 3 months after presentation with COVID-19 was preserved. Despite most of the patients continued to experience persistent fatigue, adrenal and thyroid alterations were not found [62]. Their study cohort had a normal response to Synacthen (tetracosactide, an ACTH analog), irrespective of the severity of COVID-19, their antibody status, or whether they had received dexamethasone [62]. However, until adequate studies are available assessing adrenal function in larger populations experiencing long COVID, adrenal insufficiency should be considered as a part of differential diagnosis as a cause of persistent chronic fatigue, dizziness, hypotension, and nausea, especially in patients with history of prolonged high dose steroid use. Primary adrenal insufficiency due to adrenal hemorrhage or infarction is life threatening and therefore we suggest performing early adrenal axis testing for COVID-19 patients with clinical suspicion of sudden adrenal insufficiency.

### Thyroid gland

Currently, direct or indirect effects of SARS-CoV-2 on thyroid function have not been established. Data on the effects of COVID-19 on the thyroid is very scarce and past studies on SARS-CoV have been contradictory and inconclusive due to alternative explanations. For example, Ding et al. did not detect SARS-CoV expression in the thyroid [45]. Although Gu et al. found SARS genomic sequence positive lymphocytes and monocytes in the vessel of the thyroid gland from a SARS autopsy, viral dissemination of immune cells to various organs cannot be ruled out [63]. On a postmortem study of SARS-CoV-2 patients, Hanley et al. found two (22%) of nine patients with chronic inflammation in the thyroid with follicular epithelial cell disruption, a finding of unknown significance [57]. In addition to direct damage to the thyroid gland, an immune mechanism of thyroid damage is also plausible considering that SARS-CoV-2 is able to induce systemic organ damage via the inflammatory-immune pathway. A report by Knack et al describes a case of a patient who developed Hashimoto’s thyroiditis after remission from SARS-CoV-2 supporting the hypothesis of a possible relationship of autoimmune disease being triggered by the virus [64]. Two other cases of autoimmune hyperthyroidism after COVID-19 infection have also been reported, one with a previous history of Graves’ disease in remission and another with no previous known thyroid disease [65].

In active, severe COVID-19, studies have found that levels of total triiodothyronine (TT3) and thyroid stimulating hormone (TSH) were lower in COVID-19 patients than the healthy group, especially in severe cases [66]. This might be partially explained by nonthyroidal illness syndrome. Given the resemblance between the symptoms of long COVID and hypothyroidism, it is a valid concern that the thyroid axis might have been affected. However, in a study where adults without a known thyroid disorder and COVID-19 were followed up for long COVID, most abnormal thyroid function tests in acute COVID-19 resolved, and incident thyroid dysfunction was rare [67]. In another observational cohort study on 334 COVID-19 patients, most COVID-19 patients were euthyroid (86.6%) on admission [68]. They noted TSH and free T4 (N = 185) was lower than baseline in keeping with non-thyroidal illness, but on follow-up for a median of 79 days, most patients became euthyroid again, however, the study did not follow-up patients for symptoms of long COVID [68]. In conclusion, insufficient data exist to suggest thyroid involvement as the cause of fatigue in CFS with long COVID.

### Pancreas

It certainly is conceivable that the cytotoxic effects of COVID-19 affect pancreatic islet function and trigger development of diabetes mellitus. There is a paucity of mechanistic case studies on this topic but there are 2 recent excellent review articles [69], [70], [71].

### Conclusion and future directions

Patients recovering from COVID-19 who continue to get affected by debilitating symptoms are likely to need long term support through individually tailored cardiopulmonary and psychological rehabilitation programs. Clinicians need to be aware about the likely long-term complications and their diagnostic assessments to help identify any occult problems requiring additional help. Endocrinological evaluations should be considered as part of the armamentarium in the management of such individuals with diligent cognizance about the involvement of the HPA axis, adrenal and thyroid function. Managing potentially worsening metabolic health is also important, considering that many individuals including patients infected with SARS-CoV-2 have gained weight during the Covid-19 pandemic [72]. As pointed out by Chrousos and Kaltsas for patients with post-SARS sickness syndrome manifestations and hypocortisolism, the challenge for patients with post-SARS-CoV-2 syndrome will be when to treat them with hormones [73]. Most of these patients may have an adaptive response of the HPA axis after major stress analogous to patients with nonthyroidal illness syndrome which we typically do not treat with hormone replacement.

## Declaration of Competing Interest

The authors declare that they have no known competing financial interests or personal relationships that could have appeared to influence the work reported in this paper.
